# Red‐blood‐cell manufacturing methods and storage solutions differentially induce pulmonary cell activation

**DOI:** 10.1111/vox.12911

**Published:** 2020-03-12

**Authors:** Mathijs R. Wirtz, Ruqayyah J. Almizraq, Nina C. Weber, Philip J. Norris, Suchitra Pandey, Philip C. Spinella, Jennifer A. Muszynski, Jason P. Acker, Nicole P. Juffermans

**Affiliations:** ^1^ Laboratory of Experimental Intensive Care and Anesthesiology Amsterdam UMC University of Amsterdam Amsterdam the Netherlands; ^2^ Department of Intensive Care Medicine Amsterdam UMC University of Amsterdam Amsterdam the Netherlands; ^3^ Laboratory Medicine and Pathology University of Alberta Edmonton AB Canada; ^4^ Blood Systems Research Institute San Francisco CA USA; ^5^ Departments of Laboratory Medicine and Medicine University of California San Francisco CA USA; ^6^ Department of Laboratory Medicine University of California San Francisco CA USA; ^7^ Blood Centers of the Pacific (member of Blood Systems) San Francisco CA USA; ^8^ Department of Pediatrics Division of Critical Care Washington University in St Louis St Louis MO USA; ^9^ Department of Pediatrics Division of Critical Care Medicine Nationwide Children's Hospital Columbus OH USA; ^10^ Centre for Innovation Canadian Blood Services Edmonton AB Canada

**Keywords:** blood processing, cytokine and chemokine production, extracellular vesicles, mechanical ventilation, thrombin generation

## Abstract

**Background and Objectives:**

Red‐blood‐cell (RBC) transfusion is associated with lung injury, which is further exacerbated by mechanical ventilation. Manufacturing methods of blood products differ globally and may play a role in the induction of pulmonary cell activation through alteration of the immunomodulatory property of the products. Here, the effect of different manufacturing methods on pulmonary cell activation was investigated in an *in vitro* model of mechanical ventilation.

**Materials and Methods:**

Pulmonary type II cells were incubated with supernatant from fresh and old RBC products obtained via whole blood filtration (WBF), red cell filtration (RCF), apheresis‐derived (AD) or whole blood‐derived (WBD) methods. Lung cells were subjected to 25% stretch for 24 h. Controls were non‐stretched or non‐incubated cells.

**Results:**

Fresh but not old AD products and WBF products induce lung cell production of pro‐inflammatory cytokines and chemokines, which was not observed with WBD or RCF products. Effects were associated with an increased amount of platelet‐derived vesicles and an increased thrombin‐generating capacity. Mechanical stretching of lung cells induced more severe cell injury compared to un‐stretched controls, including alterations in the cytoskeleton, which was further augmented by incubation with AD products. In all read‐out parameters, RCF products seemed to induce less injury compared to the other products.

**Conclusions:**

Our findings show that manufacturing methods of RBC products impact pulmonary cell activation, which may be mediated by the generation of vesicles in the product. We suggest RBC manufacturing method may be an important factor in understanding the association between RBC transfusion and lung injury.

## Introduction

Red‐blood‐cell (RBC) transfusion has repeatedly and consistently been associated with acute lung injury in observational studies [1, 2]. This association seems particularly clear in the critically ill and injured patient populations [3]. The mechanism is not known, but biologic effects of differences between manufacturing methods of RBCs may play a role. We have shown that the use of different filtration methods or storage solutions influences the amount of extracellular vesicles (EVs) in the RBC product, which was associated with monocyte activation [4]. Also, although the mechanism is not clear, an observational study found that transfusion of units produced using a whole blood leucoreduction method was associated with mortality, whereas transfusion of red cell filtered units was not [5].

Possibly, the risk of RBC transfusion inflicted lung injury is synergistic with mechanical ventilation, as the incidence of lung injury following transfusion is the highest in patients on mechanical ventilation [6]. In line with this, high peak pressure during mechanical ventilation is a risk factor for transfusion‐related acute lung injury (TRALI) in observational studies [7]. Additionally, we showed that the interaction between mechanical ventilation and transfusion in the induction of lung injury occurs even after a relatively short period of ventilation [8]. The observation that mechanical ventilation seems synergistic with transfusion in the induction of lung injury is relevant, as the critically ill and surgical patients receive the majority of the hospital blood supply. As the purpose of mechanical ventilation is to improve oxygen delivery, there is a need to understand the impact of RBC manufacturing methods on the occurrence of lung injury. Also, while a single RBC transfusion may cause some lung injury but not the full blown clinical TRALI syndrome, we hypothesize that every transfusion represents a pulmonary ‘hit’. Together with other ‘hits’, such as mechanical ventilation, a patient may at some point reach a critical threshold and go on to develop overt acute respiratory distress syndrome (ARDS). Therefore, it is important to identify potential causative factors in blood products that interacts with lung cells.

## Materials and methods

### Pulmonary cells

Human type II alveolar cells (A549 cells) were grown in RPMI medium, supplemented with 10% fetal bovine serum, 1% penicillin/streptomycin, 1% amphotericin, 0·43% L‐glutamine and 1% HEPES. The cells were cultured on collagen I‐coated flexible‐bottomed culture wells (35‐mm diameter; Bioflex, Flexcell International, Burlington, VT, USA) to at least 90% confluence for 48 h, since A549 cells have the ability to form adherent junctions and tight junctions when grown to confluence.

### Cyclic stretch

A549 cells were incubated with supernatant from fresh (day 4–5) and old (day 41–42) RBC products and subjected to 25% stretch at a frequency of 12 cycles/min (0.2 Hz) using a computer‐driven FX‐3000 Flexcell strain unit (Flexcell International) for 24 h at 37°C in the presence of 5% CO_2_. These parameters reflect mechanical ventilation with high tidal volumes and are based on previously published methods [9] and pilot studies. The device creates a vacuum below the flexible membrane which deforms the cells grown on top of the membrane to mimic the stress on pulmonary cells during mechanical ventilation. Control cells were either cultured on the flexible membranes, but not subjected to stretch; or subjected to stretch, but not incubated with supernatant of RBC products. Supernatant of the stretched and non‐stretched cells was collected after 24 h of (cyclic stretch) exposure.

### Blood product preparation

All blood donors provided signed informed consent at the time of donation. RBC units were produced using whole blood filtration (WBF), red cell filtration (RCF), apheresis‐derived (AD) and whole blood‐derived (WBD) methods (*n* = 8 per method, see supplemental Fig. S1).

WBF Method: Whole blood was collected with 70 mL citrate‐phosphate‐dextrose (CPD)‐anticoagulant (in a1:7 ratio), after which it was cooled to 1–6°C and leucoreduced by filtration within 48 h. Filtered units were centrifuged at 4552× ***g*** for 6 min to separate the blood components. An automated extractor was used to extract plasma and saline‐adenine‐glucose‐mannitol (SAGM) was added to the RBCs. RCF Method: Whole blood was collected with 70 ml of CPD‐anticoagulant (in a1:7 ratio) and cooled to 18–24°C overnight. Whole blood units were then centrifuged at 3493× ***g*** for 11 min and separated into blood components (plasma, RBCs and buffy coat). SAGM was added to the RBCs before leucoreduction by filtration within 24 h of stop bleeding time. AD Method: RBCs were collected using apheresis cell separators (Trima Aceel® Apheresis System,Terumo BCT; Software 6.0.6; Trima Accel 80500 kit) with 70 ml of anticoagulant citrate‐dextrose‐solution‐A (ACD‐A, in a 1:7 ratio) and 200 ml additive solution‐3 (AS‐3). During collection, RBC units were filtered (leucoreduced). WBD Method: Whole blood was collected into blood collection sets (Fenwal 4R1587P Flex Triple, WB 500 ml) with 70 mL of CPD‐anticoagulant (in a 1:7 ratio), stored at room temperature within 8 h of stop bleeding time and centrifuged at 5895xg for 8 min at 1–6°C. Plasma was extracted, 110 ml of AS‐1 was added and the RBCs were leucoreduced. We compared these manufacturing method combinations as they are most commonly used worldwide [10, 11]. A detailed description of manufacturing methods was published previously [4].

### Assays

Levels of IL‐6 and IL‐8 were measured using commercially available enzyme‐linked immunosorbent assay (ELISA) according to the manufacturer’s instructions (R&D Systems, Abingdon, UK). Thrombin generation was measured in supernatant of all blood products using the Calibrated Automated Thrombogram® assay. A total of 80 µl of supernatant of the RBC products were added to each well. Coagulation was triggered by recalcification without the addition of tissue factor, 4 μm phospholipids and 417 μm fluorogenic substrate Z‐Gly‐Gly‐Arg‐AMC (Bachem, Bubendorf, Switzerland). As no tissue factor was added, EVs are considered to be the drivers of coagulation [12]. Fluorescence was monitored using a Fluoroskan Ascent fluorometer (ThermoLabsystems, Helsinki, Finland). Lag time (minutes), time to peak (minutes), peak thrombin (nm), velocity index (nM/minute) and endogenous thrombin potential (ETP, nm⋅min), were derived by Thrombinoscope software (Thrombinoscope BV).

### Immunofluorescent staining and microscopy

At the end of the stretching episode, lung cells were fixed on the flexible membranes using 4% formalin. They were permeabilized using 0.01% Triton‐X 100 and stained using Rhodamine phalloidin (Thermo Fisher Scientific, Waltham, MA, USA), followed by staining with E‐cadherin‐ phycoerythrin (PE, Miltenyi Biotec, Bergisch Gladbach, Germany) and a secondary antibody AlexaFluor 488‐conjugated goat anti‐mouse IgG (Thermo Fisher Scientific). The second antibody was used because Rhodamine has a high fluorescence intensity and an overlapping emission spectrum with the PE‐labelled E‐cadherin we had used. After the cells were washed with PBS, the cover glasses were mounted using ProLong Gold‐antifade reagent with 4,6‐diamidino‐2‐phenylindole (DAPI; Thermo Fisher Scientific). Fluorescent images were acquired using a Leica DM‐RA Microscope, coupled to a CCD camera (Leica Microsystems, Wetzlar, Germany) equipped with Image‐Pro Plus software (Media Cybernetics, Rockville, MD, USA).

### QNano assay for extracellular vesicle concentration and size‐profiling

Quantification and size characterization of EVs in RBCs were measured using a tunable resistive pulse sensing instrument (TRPS/qNano system; IZON Science Ltd, Medford, MA, USA) as previously described in detail [13, 14]. Two different nanopores (NP200 and NP400) were used to target EVs <1.0 µm in size using a standard stretch range (43–47 mm). Carboxylate polystyrene calibration particles (CPC200; IZON Science Ltd) were used with the NP200 to characterize EVs less than 200 nm in diameter, while CPC500 (IZON Science Ltd.) were used with the NP400 to calibrate for EVs ≥200 nm. Supernatant samples were diluted with electrolyte solution (Measurement Electrolyte, IZON Reagent kit, RK1) and the sample dilution was adjusted to target a particle rate of 1000–2000/min. Samples were filtered using a 0.80 μm Millex syringe filter (Merck Millipore Ltd) before being analysed with NP400 or NP200 as recommended by the manufacturer. Samples and calibration particles measurements were run under the same conditions and at least 1000 particles were recorded with two different standard pressure ranges (1 unit = 1 mbar). Data obtained were analysed using IZON Control Suite software Version 3.3.

### Flow cytometry assay for extracellular vesicle phenotyping and quantification

Extracellular vesicles phenotyping was performed using a flow cytometer (FC) as previously described [15, 16]. Briefly, 20 μl of the supernatant of each RBC product was stained with the following linage‐specific monoclonal antibodies to identify the cell of origin of EVs: CD41a‐PerCP‐Cy5.5, CD142‐APC, CD66b‐PE, CD144‐BV421, CD235a‐FITC, CD3‐FITC, and CD14‐PE‐Cy7 (Biolegend, San Diego, CA, USA), and CD16‐ECD, CD19‐PerCP‐Cy5.5 and CD62P‐APC (BD Biosciences, Chatham, NJ, USA). Stained samples were run through an LSRII flow cytometer (BD Biosciences); sufficient events were collected to provide approximately ≥5·000 gated EV events. An AbC Anti‐Mouse Bead Kit (Life Technologies, Carslbad, CA, USA) was used to set the compensation along with the single‐stained compensation control. Small size beads, ranging from 0.2 to 1 μm (Megmix‐Plus SSC beads, Biocytex, Marseille, France), were used to generate the EV gate and to further classify them based on their size (only EVs ≤ 1.0 µm in diameter were analysed). BD TruCOUNT tubes (BD Biosciences) were used to obtain the absolute number of EVs/μL. Data were analysed using FlowJo v10.

### Statistical analysis

Comparisons between RBC product groups were analysed using ANOVA with a Dunnett’s post‐test for multiple comparisons. Outliers were removed from the dataset if values exceeded the mean ± 2 times the standard deviation. Paired t‐tests were used to identify significant differences in cytokine and chemokine production and TG potential for fresh and old RBCs. Graphs were made using Prism 7.00 (GraphPad Inc., La Jolla, CA, USA). Statistical analyses were performed using IBM SPSS Statistics 24.0 (Armonk, NY, USA). *P*‐values less than 0·05 were considered significant.

## Results

### Effect of incubation of non‐stretched pulmonary cells with RBC products on cytokine and chemokine production

**Fig. 1:**
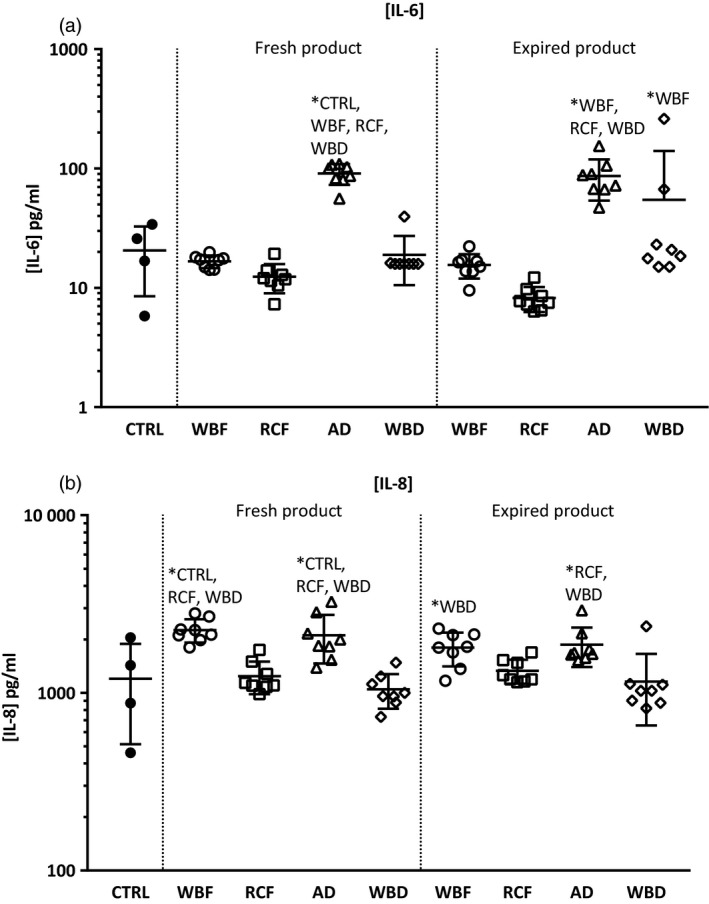
Cytokine and chemokine production in non‐stretched pulmonary cells incubated with fresh and old differently manufactured blood products. IL‐6 and IL‐8 production in non‐stretched pulmonary cells incubated with fresh and old differently manufactured blood products. AD, Apheresis‐Derived product; CTRL, Control (non‐stretched, non‐incubated cells); RCF, Red Cell Filtered product; WBD, Whole Blood‐Derived product; WBF, Whole Blood Filtered product. Values are presented as means and standard deviation. *P < 0·05 between groups.

The amount of pulmonary cell activation differed by manufacturing method. Incubation with fresh AD products significantly increased lung cell production of IL‐6 and IL‐8 compared to non‐incubated controls and the RCF and WBD products. Incubation with old AD products increased levels of IL‐6 production compared to the other blood products and IL‐8 production was significantly higher compared to RCF and WBD products. Incubation with WBF products increased IL‐8 production compared to other products (Fig. 1). The RCF products, both fresh and old, induced the least cytokine and chemokine production in resting lung cells.

### Effect of incubation of stretched pulmonary cells with RBC products on cytokine and chemokine production

**Fig. 2:**
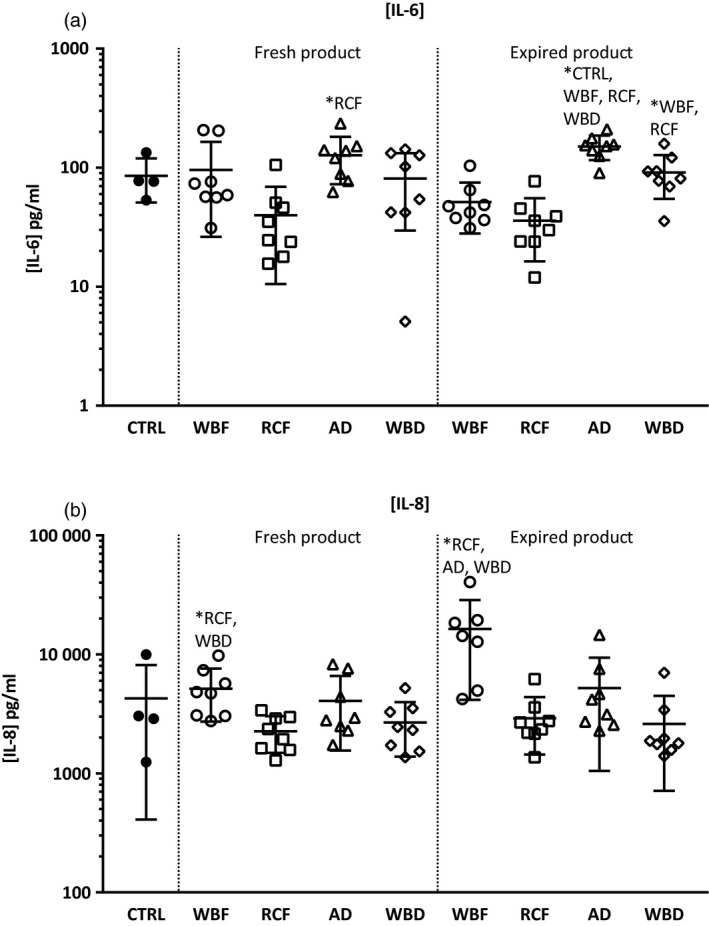
Cytokine and chemokine production in stretched pulmonary cells incubated with fresh and old differently manufactured blood products. IL‐6 and IL‐8 production in stretched pulmonary cells incubated with fresh and old differently manufactured blood products. AD, Apheresis‐Derived product; CTRL, Control (non‐stretched, non‐incubated cells); RCF, Red Cell Filtered product; WBD, Whole Blood‐Derived product; WBF, Whole Blood Filtered product. Values are presented as means and standard deviation. *P < 0·05 between groups.

Stretching of lung cells induced more cytokine and chemokine production from pulmonary cells compared to un‐stretched cells. Incubation with fresh RBC products did not further augment pulmonary cell activation due to stretching. Pulmonary cell activation induced by stretching was further augmented following incubation with old AD products, resulting in higher IL‐6 levels when compared to stretched controls and the other blood products. The WBF products, both fresh and old, induced more IL‐8 production when compared to the other products. Again, incubation with fresh and old RCF products induced the least cytokine and chemokine production (Fig. 2).

### Effect of stretching and incubation with RBC products on cytoskeleton

**Fig. 3:**
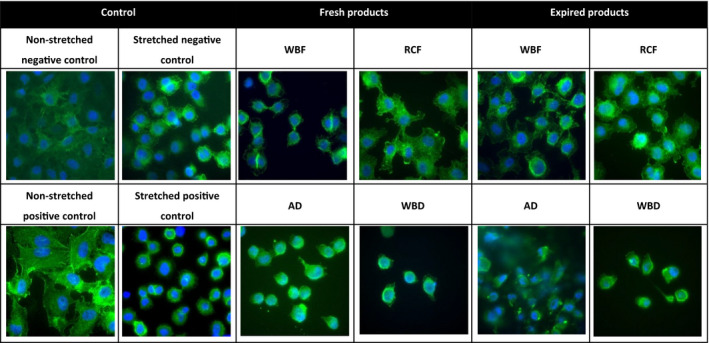
Immunofluorescent staining of stretched pulmonary cells incubated with fresh and old differently manufactured blood products. Immunofluorescence staining of E‐cadherin (AlexaFluor488, green) and cell nuclei (DAPI, blue) in pulmonary epithelial (A549) cells. Fluorescent images were acquired using a Leica DM‐RA Microscope (magnification 100x, oil‐immersion objective), coupled to a CCD camera equipped with Image‐Pro Plus software. Negative control, Not incubated with blood product. Positive control, Spiked with TNF‐α, not incubated with blood product. AD, Apheresis‐Derived product; RCF, Red Cell Filtered product. WBD, Whole Blood‐Derived product; WBF, Whole Blood Filtered product.

Stretching of pulmonary cells and incubation with blood products caused cytoskeleton alterations (Fig. 3), including a loss of intercellular adherent junctions. Incubation of stretched cells with blood products further aggravated loss of tight junctions compared to non‐incubated stretched controls, with subsequent rounding of the cells. Incubation with AD and WBD products appear most harmful, whereas following incubation with the RCF product, the intercellular borders remain more intact.

### Extracellular vesicles in products

**Fig. 4:**
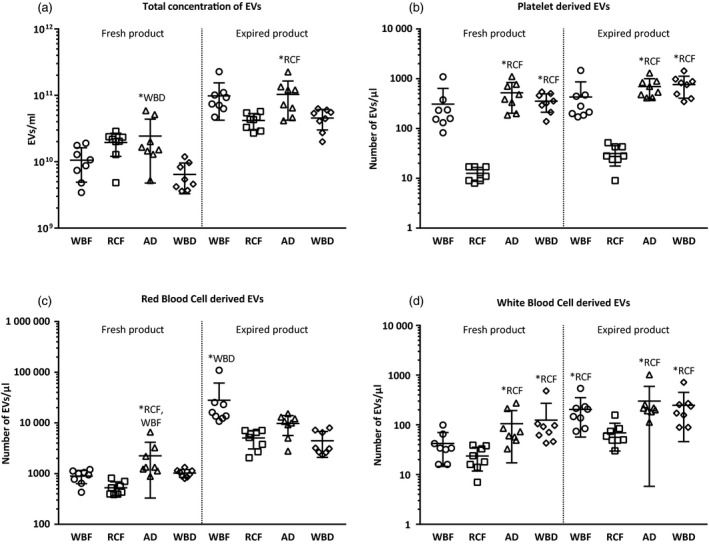
Extracellular vesicles in differently manufactured blood products. AD, Apheresis‐Derived product; RCF, Red Cell Filtered product; WBD, Whole Blood‐Derived product; WBF, Whole Blood Filtered product. Values are presented as means and standard deviation. (a) Total concentration of extracellular vesicles was measured in fresh and old differently manufactured blood products using QNano Assay. (b) Platelet‐derived (CD41a) extracellular vesicles were measured in fresh and old differently manufactured blood products using Flow Cytometry Assay. (c) RBC‐derived (CD235a) extracellular vesicles were measured in fresh and old differently manufactured blood products using Flow Cytometry Assay. (d) WBC cell‐derived (CD19+, CD14+, CD16 + and CD3 + pooled together) extracellular vesicles were measured in fresh and old differently manufactured blood products using Flow Cytometry Assay. *Statistical significant differences between groups.

AD products showed higher levels of EVs, derived from all cell lines, compared to some of the other products. The amount of platelet‐ and WBC‐derived vesicles was higher in WBD products compared to RCF products (Fig. 4b‐d). After storage, EV levels in the supernatant of all RBC products increased significantly (Fig. 4a). WBF products showed a particular rise in RBC‐ and WBC‐derived vesicles (Fig. 4c). Overall, the RCF product showed the lowest levels of platelet‐ and WBC‐derived vesicles, even after storage.

### Thrombin‐generating potential of products

**Fig. 5:**
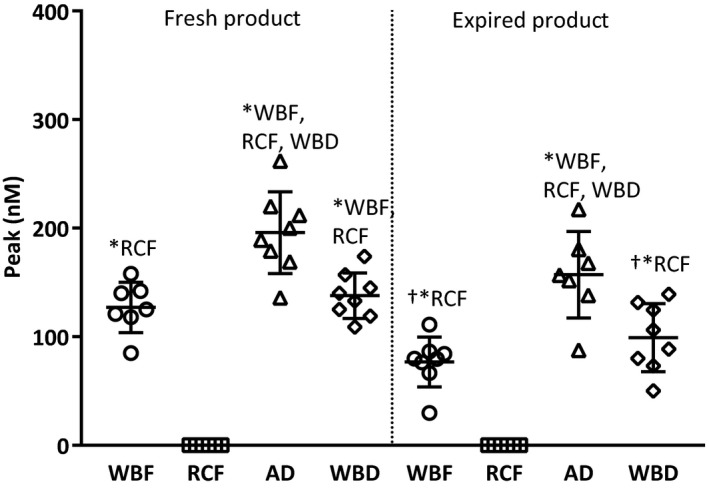
Thrombin‐generating potential of differently manufactured blood products. AD, Apheresis‐Derived product; RCF, Red Cell Filtered product; WBD, Whole Blood‐Derived product; WBF, Whole Blood Filtered product. Values are presented as means and standard deviation. *Statistical significant differences between groups.

Thrombin‐generating (TG) potential in the old products was significantly lower in comparison to the fresh products. AD products induced the highest thrombin peak compared to the other products. RCF products did not show any thrombin‐generating potential at all (Fig. 5).

## Discussion

Recently, it has become clear that manufacturing methods influence the *in vitro* characteristics of RBC products [15, 17], but little is known about associated functional outcomes. Here, we investigated the impact of manufacturing methods of RBC products on host response of resting lung cells as well as in a model of mechanical ventilation. The main findings are: (1) manufacturing method influences the amount of pulmonary cell activation induced by RBC supernatant, (2) increased pulmonary cell activation by AD products was associated with increased levels of EVs derived from RBCs and platelets and a high thrombin‐generating capacity, (3) RCF products are the least potent in activating pulmonary cells and have the lowest thrombin generation capacity and (4) overall, old products do not activate pulmonary cells more than fresh products. These results suggest that RBC manufacturing method may be important in mediating the association between RBC transfusion and lung injury.

The impact of manufacturing methods on the interaction between transfusion and lung cells may be consistent with epidemiological data [18]. Estimates of TRALI incidence vary widely across nations, with differences between Europe and the United States. In Europe, TRALI incidence is approximately 0·8 per 100 000 transfused blood products [18] and in the United States, TRALI incidence is 1·4 per 100 000 transfused blood products [19]. Obviously there are differences in reporting of cases and TRALI classification. However, different manufacturing methods used on the different continents may also have played a role. In Europe, mainly RCF products are used [11], whereas in the United States, AD, WBF and WBD products are primarily used.

Although lung cells normally are not exposed to blood products, during acute lung injury (including ventilation induced lung injury), there is disruption of the endothelial cell wall [20], which results in leakage of intravascular constituents to the parenchyma of the lungs. Incubation of lung cells with blood products induces an inflammatory host response, characterized by increased IL‐6 and IL‐8 production, as well as with alterations in the cytoskeleton. Manufacturing methods influence this interaction. WBF products and AD products induce the highest pulmonary cell inflammation, and incubation with RCF products the lowest. The combination of mechanical ventilation and incubation with supernatant of RBC products potentiates lung cell injury, including a severe loss of cytoskeleton. This occurred especially when AD products were used and to lower amounts when WBD products were used, but not when WBF or RCF products were used. The interaction between RBC products and lung injury may be mediated by EVs, as AD and WBD products containing higher levels of platelet‐derived EVs compared to the RCF product. Possibly, cell separators used for the apheresis technique results in activation of platelets with shedding of EVs, as was shown in apheresis‐derived platelet concentrates [21]. Alternatively, other components of blood product manufacturing methods, such as the preservatives, plasticizers or storage conditions, may have resulted in metabolic alterations of red‐blood‐cells and thereby have influenced lung injury [22, 23, 24]. For example, the use of di‐2‐ethylhexyl‐phthalate (DHEP), which is used in our RCF method, results in less vesicle formation compared to the use of butyryl‐trihexyl‐citrate (BTHC), which was used in the AD and WBD products [24]. Also, the amount of plasma in the various blood products in our experiment differed, with the lowest amount in the RCF products and the highest amount in the AD products, which is in agreement with previously published studies [15, 17, 25]. This may also have resulted in higher inflammatory profiles and pulmonary cell damage in the AD products, as residual plasma is linked to the occurrence of transfusion‐related acute lung injury (TRALI) [26]. Previous reports also show a pro‐inflammatory [27] and pro‐thrombotic capacity of platelet‐derived EVs [28], as well as the ability to deliver miRNAs into pulmonary cells, altering the expression of several target genes [29]. Similar interactions have been reported for RBC‐derived EVs and lung cells [30, 31]. In murine models, RBC‐derived EVs induced lung injury through priming and activation of neutrophils [32]. Thereby, we hypothesize that AD products may induce thrombin generation exacerbating lung injury through platelet‐ and RBC‐derived EVs via their membrane protein composition and through transfer of miRNAs.

An interesting finding in our study was that old products did not induce more lung injury than fresh products. Several recent clinical studies investigating the effect of storage duration of red‐blood‐cell products show no effect on clinical outcome [33], even if patients were transfused with products stored for more than 35 days [33, 34]. In addition, in a recent, very large registry cohort study, it was found that in‐hospital mortality was significantly increased when patients were treated with fresh WBF units compared to stored RCF units [5]. Together, this may point toward manufacturing method as an alternative explanation. During storage, the amount of EVs increase, which was also found in this study. In an effort to understand why EVs increase but do not seem to mediate lung injury in our model, we suggest that the bioactive properties of EVs may change over time. In line with this, membrane protein composition of the vesicles changes over time [35]. For example, phosphatidylserine (PS) exposure, which promotes thrombin generation [36], decreases in RBC EV membranes after storage [37, 38]. This is supported by the fact that TG potential of the products in our experiment was lower in the old products, even though EV levels were markedly higher. We also have shown that the phenotype and physical characteristics of the EVs change with storage time. Fresh products contained high numbers of platelet‐derived EVs, while as storage progresses products contained relatively high numbers of RBC‐derived EVs. An alternative explanation would be that vesicles are not causing pulmonary cell activation and that an as yet undefined bioactive mediator that is influenced by manufacturing method is present in RBC units.

Our study has limitations. A549 cells are adenocarcinoma cells of the lung and have the ability to differentiate when grown for many passages. We did not examine the cells to ensure that differentiation had not occurred. However, we used cells of the 2nd to 5th passage and therefore the likelihood that cells have differentiated is low. Also, we focused on the cytokine and chemokine production of type II alveolar cells. However, many more cells are involved and affected during mechanical ventilation and transfusion. This in vitro model may therefore not reflect the complexity of the host response to mechanical ventilation and transfusion in vivo. Also, we used supernatant and not the RBC cells, as this was logistically impossible. However, there are indications that it is not the cells but the supernatant which is responsible for the interaction between RBC products and lung injury [39, 40]. Also, we did not study whether the additive solution or the centrifugation method is responsible for effects. We chose to compare RBC products that are commonly used worldwide. Of note, supernatant of blood samples was incubated undiluted and without additional immune cells, which hampers extrapolation to real life. However, as experimental conditions were the same throughout, we believe that within this functional model, a difference between blood product exists in their ability to activate lung cells.

In conclusion, we have shown that commonly used RBC manufacturing methods influence the amount of pulmonary cell activation induced by RBCs in an *in vitro* model of mechanical ventilation, associated with EV concentration and TG potential. In particular, AD products seem more detrimental to pulmonary cells, whereas RCF products seem the least active. Old products are not more injurious then fresh products. Whether blood banks need to align their manufacturing method awaits further research.

## Funding Statement

This work was funded by the Canadian Blood Services Intramural Grant program [grant number 2015IG‐JA].

## Conflict of interests

The authors declare no conflict of interests.

## Supporting information


**Fig S1.** Blood product preparation methods.
