# Supplementary figures and images for: Red‐blood‐cell manufacturing methods and storage solutions differentially induce pulmonary cell activation
Source: Vox Sang. 2020 Mar 12;115(5):395–404. doi: 10.1111/vox.12911 (PMC7497002; doi:10.1111/vox.12911)

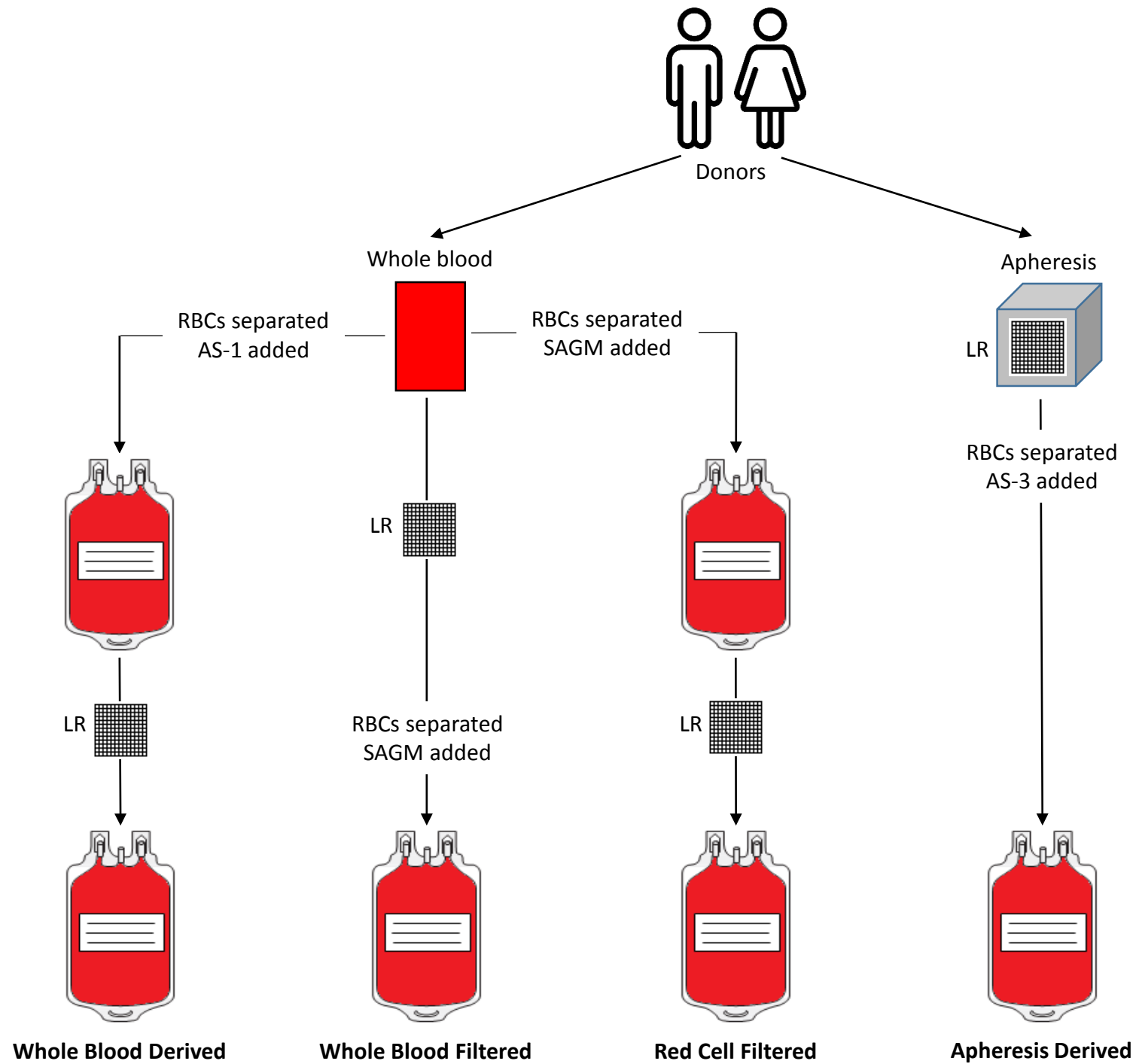

Supplement: Supplementary file 1 — Fig S1. Blood product preparation methods. [file VOX-115-395-s001.pdf]
